# Auditory Display of Fluorescence Image Data in an In Vivo Tumor Model

**DOI:** 10.3390/diagnostics12071728

**Published:** 2022-07-16

**Authors:** Sheen-Woo Lee, Sang Hoon Lee, Zhen Cheng, Woon Seung Yeo

**Affiliations:** 1 Department of Radiology, Eunpyeong St. Mary’s Hospital, College of Medicine, The Catholic University of Korea, Seoul 03312, Korea; leesw1@catholic.ac.kr; 2 Department of Radiology, University of Ulsan College of Medicine, Seoul 05505, Korea; shlee@amc.seoul.kr; 3 Molecular Imaging Program, Stanford University, San Francisco, CA 94305, USA; zcheng@simm.ac.cn; 4 Department of Content Convergence, Ewha Womans University, Seoul 03760, Korea

**Keywords:** auditory display, sonification, fluorescence imaging, hyperspectral data

## Abstract

*Objectives*: This research aims to apply an auditory display for tumor imaging using fluorescence data, discuss its feasibility for in vivo tumor evaluation, and check its potential for assisting enhanced cancer perception. *Methods*: Xenografted mice underwent fluorescence imaging after an injection of cy5.5-glucose. Spectral information from the raw data was parametrized to emphasize the near-infrared fluorescence information, and the resulting parameters were mapped to control a sound synthesis engine in order to provide the auditory display. Drag–click maneuvers using in-house data navigation software-generated sound from regions of interest (ROIs) in vivo. *Results*: Four different representations of the auditory display were acquired per ROI: (1) audio spectrum, (2) waveform, (3) numerical signal-to-noise ratio (SNR), and (4) sound itself. SNRs were compared for statistical analysis. Compared with the no-tumor area, the tumor area produced sounds with a heterogeneous spectrum and waveform, and featured a higher SNR as well (3.63 ± 8.41 vs. 0.42 ± 0.085, *p* < 0.05). Sound from the tumor was perceived by the naked ear as high-timbred and unpleasant. *Conclusions*: By accentuating the specific tumor spectrum, auditory display of fluorescence imaging data can generate sound which helps the listener to detect and discriminate small tumorous conditions in living animals. Despite some practical limitations, it can aid in the translation of fluorescent images by facilitating information transfer to the clinician in in vivo tumor imaging.

## 1. Introduction

Providing superior biological information compared with that of standard clinical imaging modalities, molecular imaging is now widely applied in order to distinguish between healthy and diseased tissue in living subjects, especially for the early detection of tumors [1]. To this end, a variety of molecular imaging approaches have been adopted. Examples include optical imaging, iron-labeled magnetic resonance imaging, as well as positron-emitter-labeled imaging, with each having their own individual strengths and weaknesses [2]. While optical imaging probes, such as luciferase or green fluorescence protein, are sensitive and safe from ionizing radiation, they are unsuitable for in vivo imaging due to photon absorption by inherent tissue chromophores or scattering [3]. Another optical technology that can improve light penetration is the near-infrared fluorescence (NIRF) probe. Advantages of NIRF imaging include increased photon penetration into tissue, decreased scattering, and wider Stokes shift, which provide images with improved sensitivity [4]. Its disadvantage, however, lies in issues encountered daily in living tissues, such as (1) autofluorescence of living tissue caused by food particles or fur mites, (2) a longer circulation time than short-lived radioisotope-labeled ligands, and (3) potential toxicity [3,4,5]. Practical solutions for some of these problems include the use of hairless hypo-pigmented mice, such as nude mice (nu/nu), that are given a thorough alcohol scrub before imaging sessions in order to remove any particles on their skin. However, these solutions are not feasible for larger mammals. 

**Figure 1:**
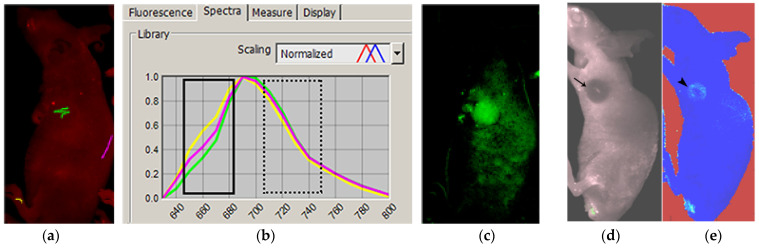
Hyperspectral data, fluorescence only, and an augmented image from parameters for auditory display. Fluorescence images were acquired under an emission filter setting from 640 to 800 [nm] in 10 [nm] increments. Hyperspectral data were overlaid on grayscale photographs to identify the ROI (**a**). Markers placed on ROIs obtained the pattern of the hyperspectral data (**b**). Spectral unmixing and overlay of the unmixed data on the grayscale images showed the tumor area with the accentuated NIRF spectrum, confirming the tumor uptake of the NIRF probe (**c**). Tumor without visible NIRF uptake on fluorescence only (**d**) was shown to have signal aided by data for auditory display (**e**).

Nevertheless, in order to facilitate a translation for human use, fluorescence imag-ing modalities have been explored in order to overcome the issues mentioned above. In-stead of obtaining single wavelength data, a multispectral method with spectral decom-position has been implemented in fluorescence imaging to provide a better chance for interrogating living biological tissue. The data obtained by fluorescence imaging units are “hyperspectral”, which features a three-dimensional data space with spatial infor-mation in the x-y plane and spectral information in the z plane at multiple consecutive narrow-band wavelengths (Figure 1).

Acquisition and analysis of this data involve spectral library processing and deconvolution, also known as multispectral imaging, to remove background signals [5]. This multispectral imaging enables the user to visualize the fluorescence spectrum of interest. Similarly, the same data can be manipulated to accentuate the signal in a different modality, i.e., in the auditory domain (as sound). This idea of presenting information using sound is called auditory display, or (complex data) sonification depending on the type of information to be presented. 

Auditory display is defined as “the use of non-speech audio to convey information” [6]. It involves the transformation of data relations into perceived relations in an acoustic signal in order to facilitate communication or interpretation. It is particularly indicated for the comprehension of data generated by ever-expanding powerful media technologies [7,8]. One of the most familiar auditory modules is the Geiger counter, which “beeps” in response to invisible radiation levels to continuously alert the operator to dangers that might have otherwise gone unnoticed [9,10]. Recently, electric encephalography (EEG) data were effectively transformed into sound [11]. Such applications capitalize on the listener’s ability to detect small changes in auditory events, or the requirement that users should have their eyes free for other tasks. The integration of auditory and visual information is also known to enhance detection performance [12]. 

In the emerging era of intraoperative imaging, the auditory display of fluorescence data may help surgeons to navigate toward potentially cancerous lesions with distinguishable sound which is hardly detected in the visual domain. In this context, the purposes of this study are: (1) to devise a means of portraying fluorescence imaging data as sound (auditory probing); (2) to test the feasibility of an effective auditory display; and (3) to determine whether this methodology allows small fluorescence signals to be enhanced. The fluorescent-labeled probe is injected into a tumor xenograft model, and the raw hyperspectral data are presented with an auditory display method that incorporates a sound synthesizer based on frequency modulation (FM). A prostate cancer model was used as a potential analog of prostate cancer metastasis to lymph nodes, based upon the idea that if this auditory display was found to be useful in a prostate cancer model, it could potentially aid the diagnosis of early tumors and locate small, tumor-laden tissues such as those of sentinel lymph node metastasis. 

## 2. Materials and Methods

### 2.1. Cells and Animal Experiments

Animal studies were approved by the Center for Animal Care and Use and performed according to institutional ethics and safety guidelines at Gachon University of Medicine and Science, Incheon, Korea (GDIRB2014-15).

A standard prostate cancer xenograft mouse model was created by injection using a 4 × 10^6^ PC3 cell line (ATCC^®^ CRL-1435) in 15 male athymic nude mice (nu/nu; 8–10 weeks old). Excluding the mice which did not develop any tumors, there were six mice in the final study group.

When a tumor reached sufficient volume, in vivo fluorescence imaging was performed. The fluorescence imaging probe Cy5.5-2-deoxyglucose was synthesized as previously described [13]. Before imaging, tumor-bearing mice were injected via a tail vein with 0.5 [nmol] of Cy5.5-2DG, diluted in 100 [µL] of normal saline. Anesthetized animals were placed within the fluorescence imaging chamber of a *Maestro* system (Perkin Elmer, MA, USA). Images were acquired using a band-pass filter from 576 to 621 [nm] (excitation) and a long-pass emission filter over 645 [nm] (640–800 [nm] window in 10 [nm] increments). The duration of each imaging session was about 5 min per mouse. 

### 2.2. Pre-Analysis of Data 

The raw data acquired from the Maestro imaging system, which contained 18-channel hyperspectral components per pixel, were visually analyzed using *Nuance* software packages (Perkin Elmer, Waltham, MA, USA). For visual demonstration purposes, selected spectral components of interest were separated from the whole data and overlaid on grayscale photographs to produce pseudo-colored images (Figure 1). Usual visual patterns were identified by first inspecting the image data for visible fluorescence uptake in the tumor area above the background signal. The spectral patterns in the ROI were graphically displayed by marking regions with different colors. Raw data were processed by spectral unmixing to confirm tumor uptake of the near-infrared probe. 

### 2.3. Data Transport and Auditory Display

For sonification, a “transport” application program was developed using the software development kit (SDK) provided by the manufacturer of the fluorescence imaging system. This program provides an image-based data navigation interface, which allows the user to access the 18-channel hyperspectral information of the raw data and probe the ROI to listen to the result of the auditory display by clicking/dragging the computer mouse. 

**Figure 2:**
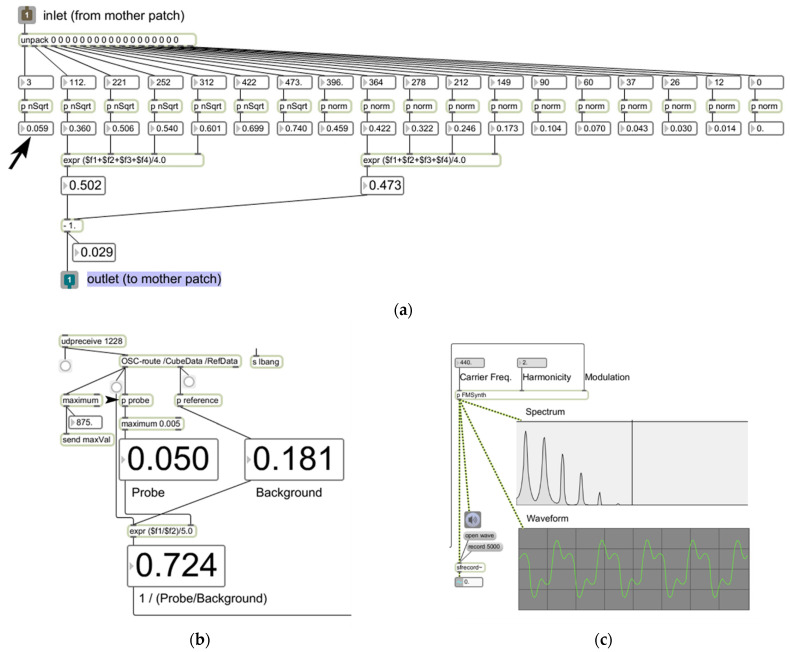
Components of the preprocessor and the sound synthesizer built on Max: (**a**) shows the algorithm to produce a target value; (**b**) illustrates the normalization process to provide the parameter value for sound synthesis; and (**c**) depicts the FM sound synthesis patch used for this study as well as the spectrum and waveform of the result of auditory display.

Data from the selected ROI are instantly transmitted to an external auditory display engine built on *Max*, a visual programming environment for multimedia programming (Cycling 74). Here, eighteen-channel hyperspectral data are processed in order to provide the key parameter for sound synthesis. First, the averaged spectrum value from four lower frequency bins (640, 650, 660, and 670 [nm]) is subtracted from that of selected mid frequency bins (710, 720, 730, and 740 [nm]) to generate a target value of the ROI (Figure 2a). This is then compared against the target value from the background, i.e., the skull (Figure 2b) to provide a “normalized” value. Finally, this normalized value is handed over to a sound synthesizer and serves as the modulation index of a two-oscillator frequency modulation (FM) method [14] (Figure 2c). The sound generated from this becomes the result of auditory display.

**Figure 3:**
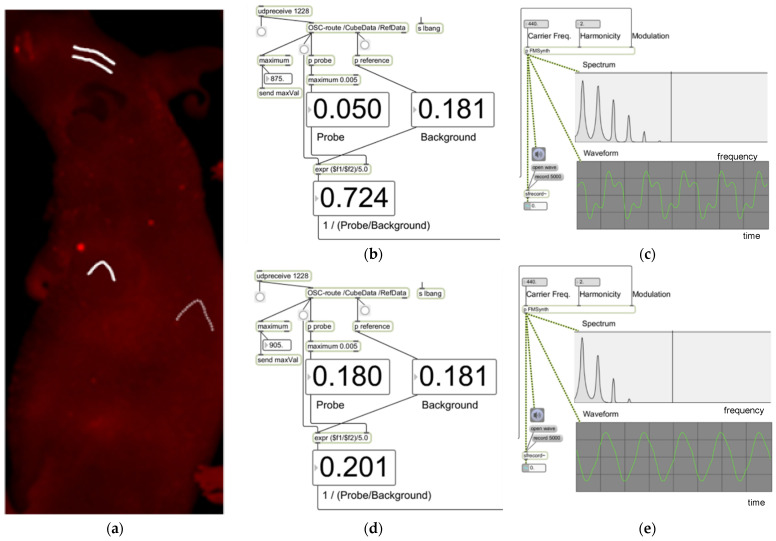
Samples of a session of auditory display. Shown in (**a**) is the navigation map (JPEG picture of a mouse during an image session). The tumor area is indicated by a single white curved line, the background skull by double white lines, and the non-tumor body by a dotted line. The rest of the figure depicts parameters for sonification as well as the spectrum/waveform of the resulting sounds from the tumor area (**b**,**c**), and those of the non-tumor area (**d**,**e**).

The in vivo results were analyzed to determine whether the sonification data allowed the tumor to be identified. Four sets of data were obtained per single ROI: sound in the discrete audible form (Supplementary Data), sound as a mapped numerical form, as well as both spectrum and waveform of the sound (Figure 3). Positive control was a mouse tail immediately after extravasated injection (Figure 1). Negative control was the skull, and the background signal from the abdomen and flank. 

For statistical analysis, the mapped numerical form (i.e., the probe-to-background ratio used to control the timbre of the sound synthesizer) was extracted from the program by scrolling and clicking on the ROIs and tabulated into an Excel spreadsheet. Result tumors versus the background from the average of three measurements were compared and analyzed using the Wilcoxon test (*p* < 0.05). To see whether this auditory display was sensitive for tumor identification, the data were converted back to images; here, the numerical data from sonification were mapped to a 0–1 scale and then transformed to images in a rainbow scale. 

## 3. Results

The positive control area showed very high auditory signals in comparison with the backgrounds (10.39 ± 0.67 vs. 0.28 ± 0.01, *p* < 0.05). The average probe signal was significantly higher for tumor areas than for the negative control (Table 1, 3.63 ± 8.41 vs. 0.42 ± 0.085, *p* < 0.05). Tumor areas also showed significantly higher values than left flanks away from the tumor or skull background value.

Sound from the tumor was perceived by the naked ear as high-pitched and less pleasant, compared with that of the tumor-free area (Supplementary Sound Files S1–S3). In addition, Figure 1 presents graphical displays of the spectrum and waveform of sonified results from six mice, comparing the sonic characteristics of tumorous areas (more high-frequency components in the spectrum, usually with a more complicated waveform) with those of a tumor-free background. 

**Figure 4:**
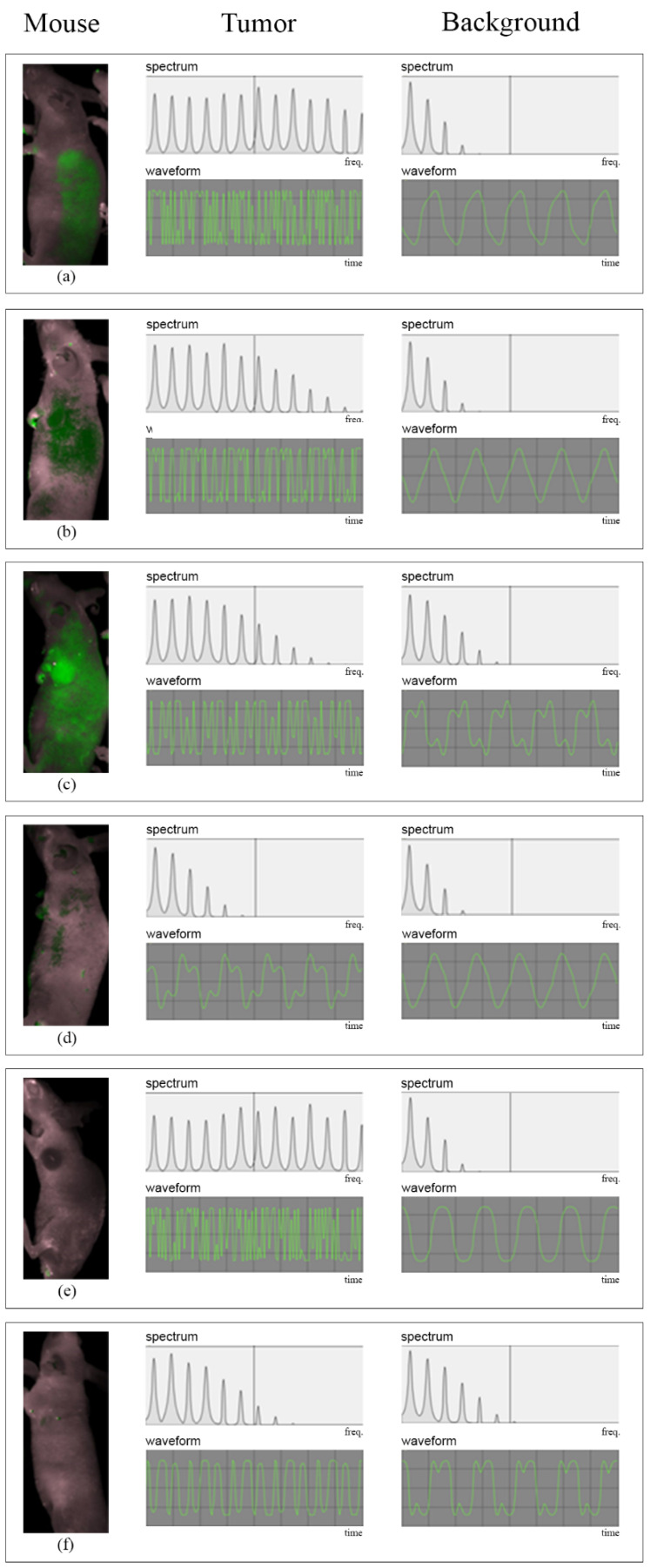
Spectrum and waveforms of the sonified results from each mouse in the experiment. The left column shows the processed NIRF images from the fluorescence imaging system on grayscale photographs of each mouse (**a**–**f**), showing variable fluorescence signals from the tumor. The middle column shows the result of auditory display from the tumor area, with the spectrum (**above**) and waveform (**below**) from the tumor. The right column shows the spectrum and waveform of the sound from the background (non-tumor) area. Compared with the background, sounds from the tumor areas of all mice featured a heterogeneous spectrum and irregular waveform.

Mice E and F showed a higher probe-to-background ratio in the tumor by the sonification. No tumor fluorescence signal was visualized on routine visual spectral unmixing, and the mice showed gross discoloration of the tumor area or small tumor size (Figure 1d or Figure 4e). The reconverted data from the sonification clearly outlined the tumor (Figure 1e).

## 4. Discussion

This study showed that an auditory display of fluorescence data can be utilized to detect and discriminate small tumorous conditions in living animals, thereby presenting the possibility of a new type of probe development for early cancer evaluation. 

We demonstrated that: (1) The feasibility of creating an auditory probe that can distinguish NIRF-emitting tumor xenografts in living mice; (2) That auditory probing allows the detection of NIRF signals from tumors not well visualized by conventional hyperspectral processing, and suggest a future probing scenario whereby auditory probes are used to locate areas with different timbres representing tumors; (3) Since it is possible to magnify a spectrum of interest in an arbitrary manner using the mapping technique, the devised technique can be used for fluorescence imaging probes other than cy5.5. Furthermore, the sonification of other imaging modalities, including those in clinical use, is also within reach, if fundamental datasets are carefully characterized and strategic mapping sequences are designed. Sonification has been applied in various settings, including medical and military settings. Its usefulness lies in situations where two different visual tasks cannot be performed at the same time, as exemplified by sonar radar on a busy ship. A comparable setting in the medical field would be the operating suite, where ambient light hides the dim fluorescence signal from tumorous conditions. While recent reports have demonstrated the concept of auditory feedback for surgical navigation, most of the studies covered the use of sound to prompt surgeons to navigate away from critical neurovascular bundles [15]. One study used audio guidance to move toward the tumor by using preoperatively drawn target outlines and fiducial markers [16]. None of the studies have used the biological information of the malignancy for real-time display. Some tumors undergo significant anatomic changes throughout the course of the disease due to edema, inflammation, or other factors. Furthermore, anatomical image-based manual contouring is potentially inaccurate [17]. Sonification of in vivo molecular information will potentially aid in the improved display of tumors or sentinel lymph nodes in the future [18,19]. 

Laser-induced fluorescence offers a sensitive, high-throughput means of imaging many aspects of molecular events in living tissues. Hyperspectral imaging is useful for overcoming the challenges posed by in vivo fluorescence but requires spectral processing to remove unnecessary background noise. For tumor detection by spectral unmixing, the process depends on choosing an area of presumed positive signal and subtracting it from the background. However, in cases with marginally positive fluorescence signals, such as tumors in a deep location in vivo, or a tumor mixture with hemorrhagic or discolored tissues, such signals can be difficult to detect. Furthermore, the acquisition of spectral library in a single inbred mouse species of homogeneous soft tissue cannot be translated into humans. We hope that the auditory display can be developed further to implement the compound imaging data in vivo. 

This study has some limitations. First, the sizes of some tumor xenografts were too small to create a large sound or a large study group. The failure of tumor development in nude mice is attributed to some immunologic competence within the species, and the cell conditions. The goal of sonification is not to find large visible tumors; although, interestingly, in mice E and F, even though the signal was not visible on fluorescence-processed images (due to small tumor size, tumor discoloration, or necrosis), the sound data did show some differences versus the background, which demonstrated the usefulness of sonification. Future studies on the detection limits of sonification for small cancerous lesions and on the pathologic basis of timbre differences are required. In addition, in vitro research using phantom studies would be useful. 

Second, although the uptake of cy5.5-glucose by tumors is specific, the mechanism involved has not been fully elucidated [13] and it may differ from naked glucose uptake by tumors. For example, it may be linked with the permeability of tumor vasculature, otherwise known as the enhanced permeability and retention (EPR) effect [20]. Further study is needed to explore the involved mechanism, and to overcome some issues during tumor harvesting, freezing, and cross-sectioning. During tissue processing, the fluorescence signals can be lost, this being related to photobleaching, meaning the destruction of the fluorophore [21]. This phenomenon is a problem during fluorescence imaging and depends on many factors, such as culture medium, the presence of oxygen or free radicals, imaging chambers, slides, the type of fluorescent agent used, and the time required for imaging [22]. Future studies should include different kinds of fluorescence probes armed with favorable wavelengths for tissue penetration, resolved toxicity issues, and less photobleaching. 

Third, the number of animals involved in the present study was small. 

Fourth, ROI size needs to be optimized, since timbres varied when scrolling over living tissue. To overcome this problem, the listener needs to detect multiple higher-timbred pixels in one area, since tumors generally have an ovoid shape with multiple similarly timbred pixels adjacent to each other.

The fifth issue is precise parameter scaling in accordance with the perceptual characteristics of the human auditory system, which is required to enhance the performance of the current system. This may involve a parameter mapping mechanism with control over user-specific features as well as optimizations of frequencies and loudness levels to ease detection. A relevant problem is the perception of the sound from a tumor compared to that of a tumor-free area. Although being blinded to the origin of the sound, the subjective reaction from the auditors may vary. Further large-scale study and training are needed to apply the sound as a sole diagnostic parameter.

Nonetheless, sonification for in vivo fluorescence imaging is a new concept that has not been previously reported in the literature. In the present study, we aimed to test the feasibility of the concept as an initial step before increasing the number of animal subjects. Further experiments with more mice and different fluorophores are necessary to elucidate the mechanism of tumor specificity, optimize the tissue handling of fluorescence-injected animals, and to validate sounds produced from tumors. 

## 5. Conclusions

In this study, we proposed that an auditory display can be applied for the facilitated transfer of in vivo tumor imaging information.

We do not yet believe that auditory display is the ultimate answer, nor do we consider it a “magic tool” by itself. While this sound-based approach was sensitive for small, masked tumors and could decrease the noise caused by autofluorescence, the listener must be trained to identify the specific timbre of tumors and may experience ear fatigue over prolonged use. In addition, the suggested probing scenario is that audio-based analysis can be used to complement traditional visual analysis methods, and that when an auditory display results in a suspicious signal, the area concerned should be examined further by additional probing, more detailed sonification, visualization, or dissection. However, even with these limitations, auditory display can aid in the translation of fluorescent images, showing the potential for enhanced cancer perception. 

## Figures and Tables

**Table 1:** The median values and range of ROI measurements of tumors and backgrounds. ROIs of quantified auditory data from tumors were higher than those of the background skull or left flank area. The values with statistical significance are highlighted in bold (*p* < 0.05).

	Skull	Tumor	Left Flank
All mice	0.34 (0.13–1.59)	4.91 (0.23–8.21)	0.55 (0.17–6.62)
Mouse A	0.31 (0.25–0.45)	2.2 (1.02–8.21)	0.98 (0.56–2.2)
Mouse B	0.47 (0.23–1.59)	6.62 (5.19–7.70)	5.18 (0.86–6.62)
Mouse C	0.66 (0.34–0.99)	5.17 (4.89–6.23)	3.8 (1.24–4.9)
Mouse D	0.41 (0.34–0.67)	6.47 (5.70–7.27)	0.51 (0.35–0.54)
Mouse E	0.21 (0.19–0.23)	0.26 (0.23–0.35)	0.24 (0.17–0.54)
Mouse F	0.71 (0.13–0.34)	0.75 (0.44–1.13)	0.26 (0.19–0.34)

## Data Availability

The data that support the findings of this study are available from the corresponding author upon reasonable request.

## Supplementary Materials

The following supporting information can be downloaded at: https://www.mdpi.com/article/10.3390/diagnostics12071728/s1, Audio files: exemplary sounds of auditory display from tumor (S1), body (S2) and skull (S3) areas, respectively.
